# Application safety evaluation of the radio frequency identification tag under magnetic resonance imaging

**DOI:** 10.1186/1475-925X-13-129

**Published:** 2014-09-04

**Authors:** Xiaolu Fei, Shanshan Li, Shan Gao, Lan Wei, Lihong Wang

**Affiliations:** Information Technology Department, Xuanwu Hospital Capital Medical University, No. 45 Changchun Street, Beijing, 100053 China; Information Management Office, Xuanwu Hospital Capital Medical University, No. 45 Changchun Street, Beijing, 100053 China

**Keywords:** RFID tags, MRI sequence, Electromagnetic interference, Imaging quality

## Abstract

**Background:**

Radio Frequency Identification(RFID) has been widely used in healthcare facilities, but it has been paid little attention whether RFID applications are safe enough under healthcare environment. The purpose of this study is to assess the effects of RFID tags on Magnetic Resonance (MR) imaging in a typical electromagnetic environment in hospitals, and to evaluate the safety of their applications.

**Methods:**

A Magphan phantom was used to simulate the imaging objects, while active RFID tags were placed at different distances (0, 4, 8, 10 cm) from the phantom border. The phantom was scanned by using three typical sequences including spin-echo (SE) sequence, gradient-echo (GRE) sequence and inversion-recovery (IR) sequence. The quality of the image was quantitatively evaluated by using signal-to-noise ratio (SNR), uniformity, high-contrast resolution, and geometric distortion. RFID tags were read by an RFID reader to calculate their usable rate.

**Results:**

RFID tags can be read properly after being placed in high magnetic field for up to 30 minutes. SNR: There were no differences between the group with RFID tags and the group without RFID tags using SE and IR sequence, but it was lower when using GRE sequence.Uniformity: There was a significant difference between the group with RFID tags and the group without RFID tags using SE and GRE sequence. Geometric distortion and high-contrast resolution: There were no obvious differences found.

**Conclusions:**

Active RFID tags can affect MR imaging quality, especially using the GRE sequence. Increasing the distance from the RFID tags to the imaging objects can reduce that influence. When the distance was longer than 8 cm, MR imaging quality were almost unaffected. However, the Gradient Echo related sequence is not recommended when patients wear a RFID wristband.

## Background

With the rapid development of the Health Information Technology (HIT) in the last 10 years, lots of people have already recognized that HIT can improve patients safety in many fields. As one of the most useful kinds of HIT, the application of Radio Frequency Identification (RFID) is attracting increasing attentions for its non-contact working mode and good traceability in the healthcare fields. The aim of using RFID tags in healthcare is to improve work efficiency and cost-effectiveness. RFID can play a key role in various kinds of applications, such as patients, medications and blood checking, inventory management, real-time patient monitoring and tracking and so on. Presently, the US government is spending about US$900,000 on the RFID system in healthcare per year. It is estimated that the overall spending on RFID tags in healthcare will surpass US$2 billion in 2018 [1–4].

Compared with bar codes, there are many advantages of RFID tags, such as working in non-contact mode, carrying more information, not easily being damaged, and suiting better than bar codes to various environmental conditions when they are resistant to moisture, crushing, and tearing. As the unique personal identification for a patient, the RFID tag must be with the patient throughout the whole medical process during his or her hospital stay to avoid incorrect identification among patients. The wristband used to fix the RFID tag can only be cut until the patient leaves the hospital. Different from bar codes based on the paper, as a coil technology, RFID tags may induce amounts of Electromagnetic Interference(EMI) into the healthcare environment. However, although several studies have shown that RFID tags may cause side effects to some critical care medical equipments, such as infusion/syringe pumps and implantable pacemakers and so on [5–7], little attention has been paid to the EMI effects caused by RFID applications in hospitals in the last few years. Considering the RFID technology widely using in healthcare without exploring EMI, studies and evaluations on patient safety affected by EMI should be made urgently [8]. Hospitals should not ignore the possibility of EMI even though the number of incidents reports is low [9].

The safety evaluation of RFID application in the hospitals should include two aspects: 1) how RFID tags affect other medical devices in the healthcare environment; 2) how RFID tags operate after being exposed to EMI. Considering Magnetic Resonance (MR) examination is a common scenario with magnetic field for patients in a hospital, we select it as the first research object in this study. The aim of this study is to assess the impact of RFID tags on MR imaging in an electromagnetic environment in hospitals and to evaluate their application safety.

## Methods

### Phantom

**Figure 1:**
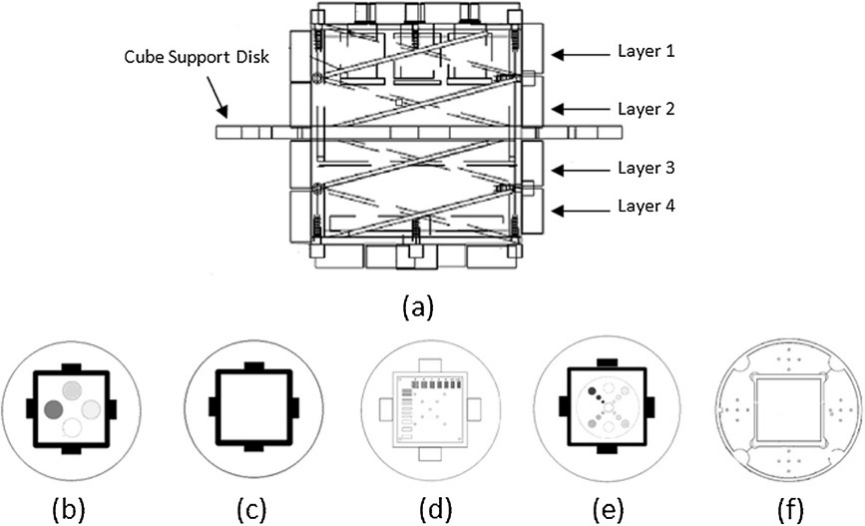
**Structure sketch of Magphan phantom.** Each layer’s image is shown in turn from left to right. The last one is the image of cube support disk which is used to obtain the geometric distortion.

This study uses the Magphan phantom designed by the US phantom laboratory (The Phantom Laboratory) to quantitatively evaluate image quality. The phantom comprises a cylindrical container made of organic glass and a test cube. The diameter of the cylindrical vessel is 20 cm, and the length is 22 cm, it contains 1 g/L Cu_2_SO_4_. Each side of the test cube is 10 cm [10]. The phantom has four layers and one support disk. Each layer is a different structure for measuring different imaging parameters (Figure 1). In this experiment, we obtained four MR imaging parameters by measuring the different layer/disk images, including (1) signal-to-noise ratio(SNR) and uniformity that are obtained from the second layer, (2) geometric distortion that is obtained from the cube support disk, and (3) high-contrast resolution that is obtained from the third layer.

### Data acquisition

#### MR scan for quantitative image quality analysis

**Figure 2:**
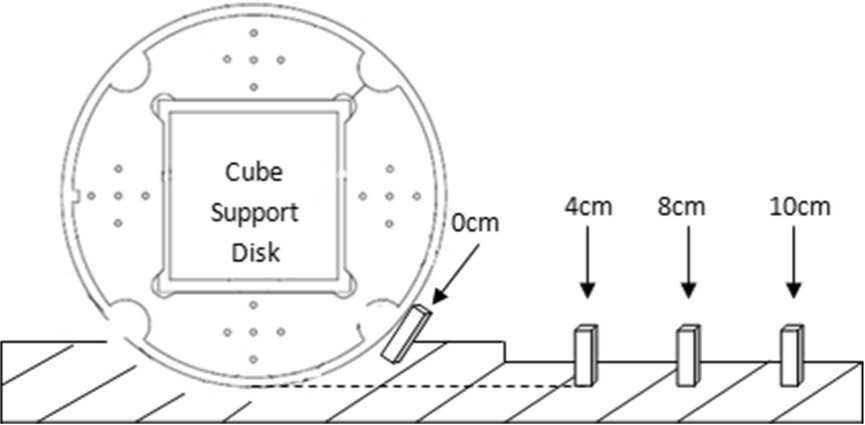
**The detailed positions of RFID tags.**

The phantom was scanned by a SIEMENS Sonata 1.5 T MRI scanner (SIEMENS Healthcare, Germany) with three typical MRI sequences [11, 12]: 1) spin-echo (SE), 2)gradient-echo (GRE), 3) inversion-recovery (IR), and in several RFID conditions: phantom without RFID tags, and phantom with RFID tags at different distances of 0 cm, 4 cm, 8 cm and 10 cm. In order to capture the little changes of artifacts and image quality parameters caused by RFID tags, the selected distances were short. The detailed position is illustrated in Figure 2. The phantom was scanned for three times in each condition. Sequence SE lasted 214 seconds, sequence GRE lasted 215 seconds and sequence IR lasted 215 seconds. The sequence protocols are listed in Table 1. Since most of the MR sequences are developed based on spin-echo or gradient-echo, we chose the basic SE and GRE sequence as the representative of MR sequence. IR is also developed based on spin-echo but it may have higher Specific Absorption Rate (SAR) value than common SE, so we also included IR in this study. All the parameters used in this study are the common parameters used in daily work to simulate the MR scan condition of patients. SAR value was calculated automatically by scanning software when assuming patient age was 25 and weight was 80 kg.

**Table 1:** **Values of sequence protocols**

Sequence	TR (ms)	TE (ms)	Flip angle (° )	FOV (mm)	SAR
SE	500	7.7	90	230	16%
GRE	194	4.78	90	230	30%
IR	8500	122	180	230	54%

Values of sequence protocols (TR, TE, Flip Angle, FOV) in the three scan sequences.

TR, repetition time; TE, echo time; FOV, field of view; SAR, specific absorption rate.

#### MR scan for usability test of RFID tags

In order to simulate the scan condition of patients as real as possible, the RFID tags were placed near the phantom at different distances: 5 cm distance referred to Region of Interest (ROI) near the wrist wearing the RFID tag; 10 cm distance referred to ROI in arms; 30 cm distance referred to ROI in waist e.g. and 70 cm distance referred to ROI in legs. Then the phantom was scanned by using SE, GRE and IR two times for each sequence. One lasted 10 minutes, and the other lasted about 30 minutes. Finally, the RFID tags were taken into a room with reading device (wireless AP).

The usability test of RFID tags contains two parts. Firstly, whether the tag could be found immediately when entering the room, reflecting whether the data packets sent from the RFID tag could be received by the reading device. Secondly, whether the identification number which was recognized by Access Point (AP) was same before and after MR scanning, demonstrating whether the data was lost or corrupted. A RFID was defined as “usable” in this experiment only if the RFID tag could be found actively by the AP and the identification number that did not change after MR scan.

In order to evaluate the heating effect caused by the long-time MR scanning on RFID tags, a temperature sensor was attached tightly to the RFID tags before and after the RFID tag was scanned. The temperature data was read and recorded as the initial and final temperature of the RFID tags respectively. Considering that the environment temperature was not very stable, the environment temperature was also measured and recorded to evaluate the effect of environment temperature on the RFID tag before every temperature measurement. The difference between the initial and final temperature was used to evaluate the heating effect.

### Data post-processing

For further post-processing and image analysis, all data sets were transferred from the MR scanner to an offline workstation (DC5800, HP, China).

#### Signal-to-noise ratio

**Figure 3:**
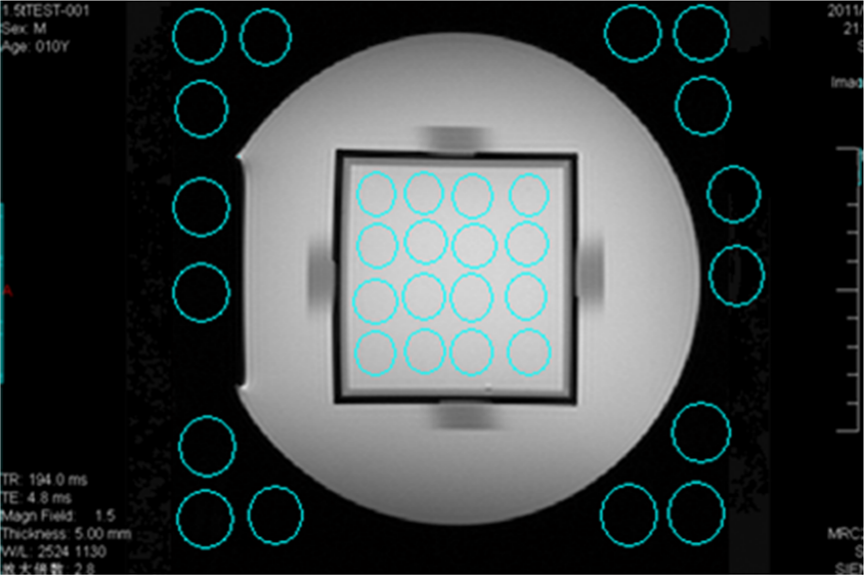
**Image used for measuring the signal-to-noise ratio; sixteen ROI in the center of the square were selected, and the mean pixel values and the standard deviation of each ROI were measured.** Then 16 ROI in the outside of cylindrical container were selected, and the mean pixel values of each were measured.

Scanning the second layer to get Figure 3, SNR was calculated by using equation (1), where *S*_0_ is equal to the difference of the mean pixel value in the center of the square and the mean pixel value in the outside of the cylindrical container, and *N*_0_ is derived from the standard deviation of the pixel value within the ROI in the center of the square.

1

#### Uniformity

**Figure 4:**
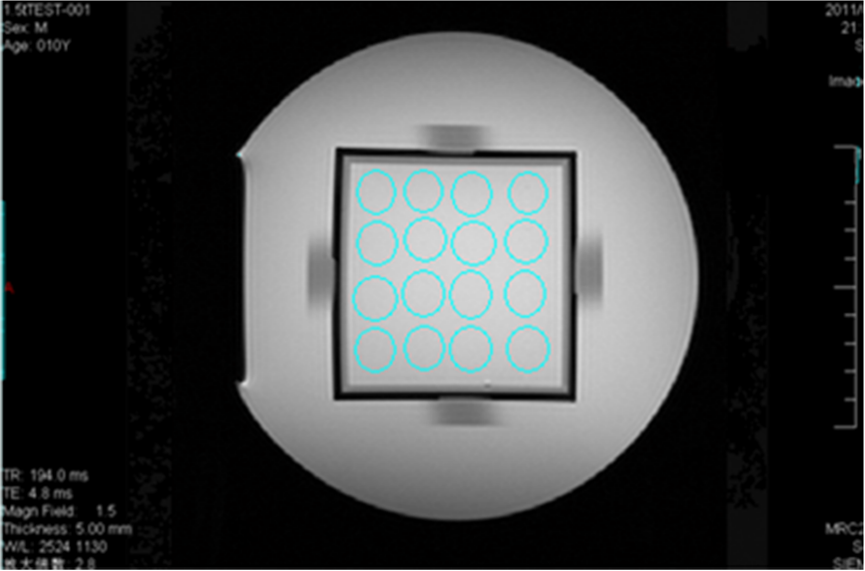
**Image used for measuring the uniformity; sixteen ROIs in the center of the square were selected, and their maximum and minimum pixel values were measured.**

Scanning the second layer to get Figure 4, uniformity was calculated by using equation (2), where *S*_max_ is the maximum pixel value of the ROI, and *S*_min_ is the minimum pixel value of the ROI.

2

#### Geometric distortion

**Figure 5:**
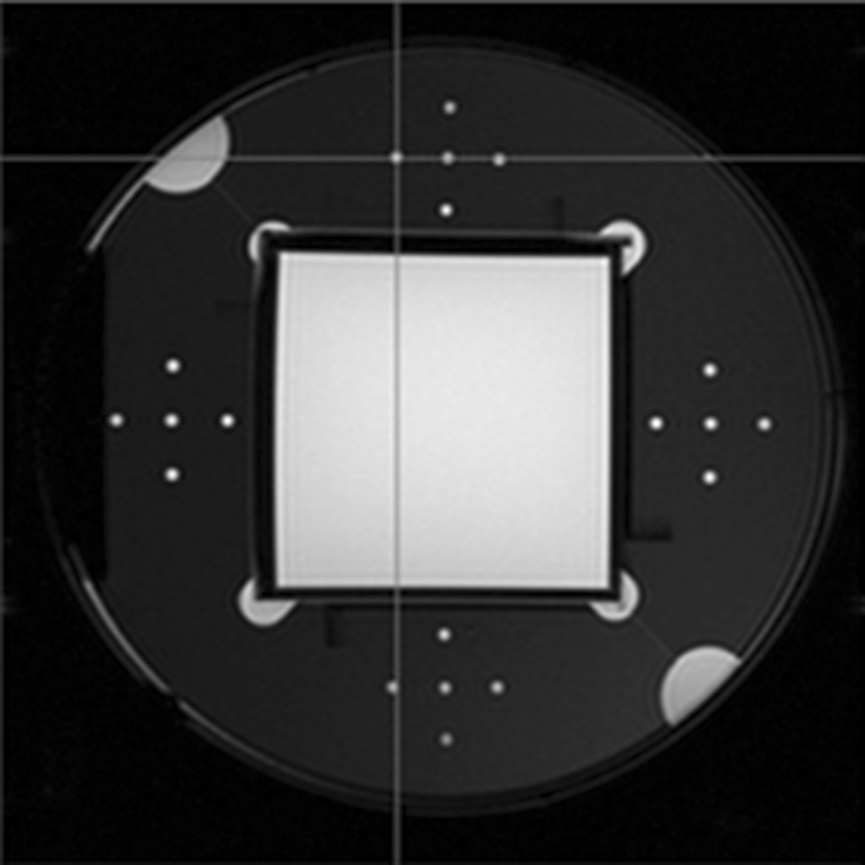
**Image used for measuring the geometric distortion.**

Scanning the cube support disk to get Figure 5, MATLAB was used for geometric distortion measurement. Twenty highlights were selected from the image and the coordinates of each highlight center were obtained. Geometric distortion was calculated by using equation (3), where *L*_*m*_ is the measured distance between each highlight center and *L*_*t*_ is the true distance between each highlight center.

3

The measurement of Geometric distortion in this experiment was used to evaluate whether the area without visible distortion was affected by RFID. To avoid the artifacts’ influence on the measurement, the white points which were obviously affected by artifacts were ruled out and were not included in the measurement.

#### High-contrast resolution

**Figure 6:**
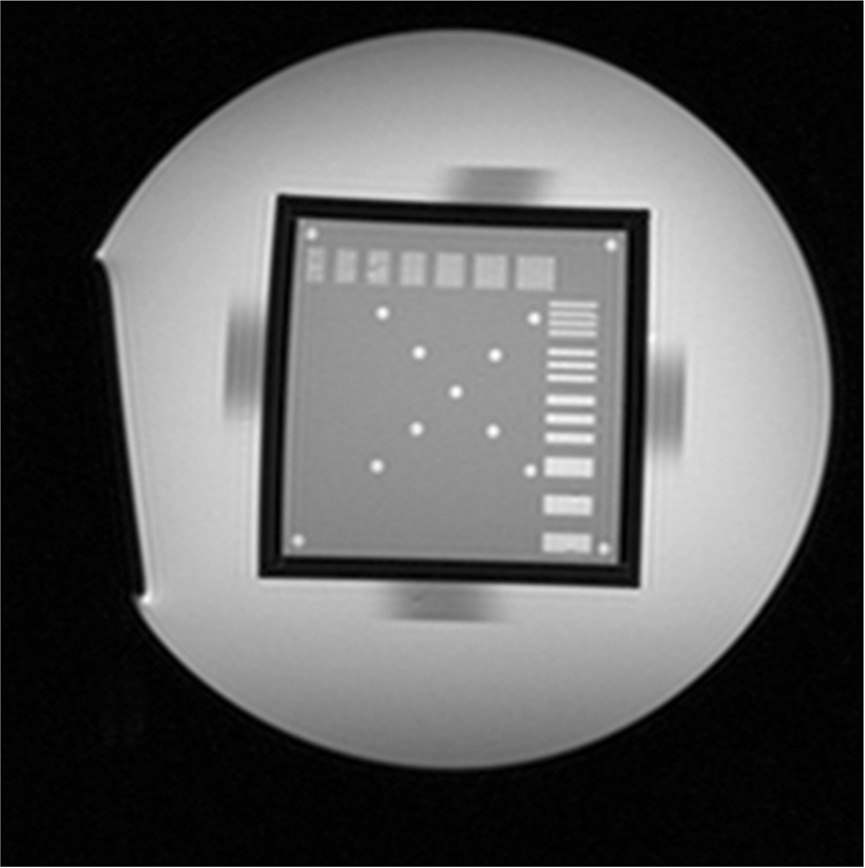
**Image used for measuring the high contrast resolution.**

Scanning the third layer to get Figure 6, the width and level of the window were adjusted until the image details could be shown clearly, and line pairs and spatial resolution could be confirmed.

### RFID tags

As the hospital Internet of Things (IOT) is developing in China, patient wristbands are no longer just as the carrier of patients’ basic information. It has been integrated into various applications in hospital as a part of IOT, such as tracking the position of the patients and babies’ anti-theft system. Compared with passive tags, active tags have longer working distance and higher identification speed. Therefore, considering the increasing applications of the active tags that are applied in medical environment, this research adopted high frequency active tags as the experimental object.

**Figure 7:**
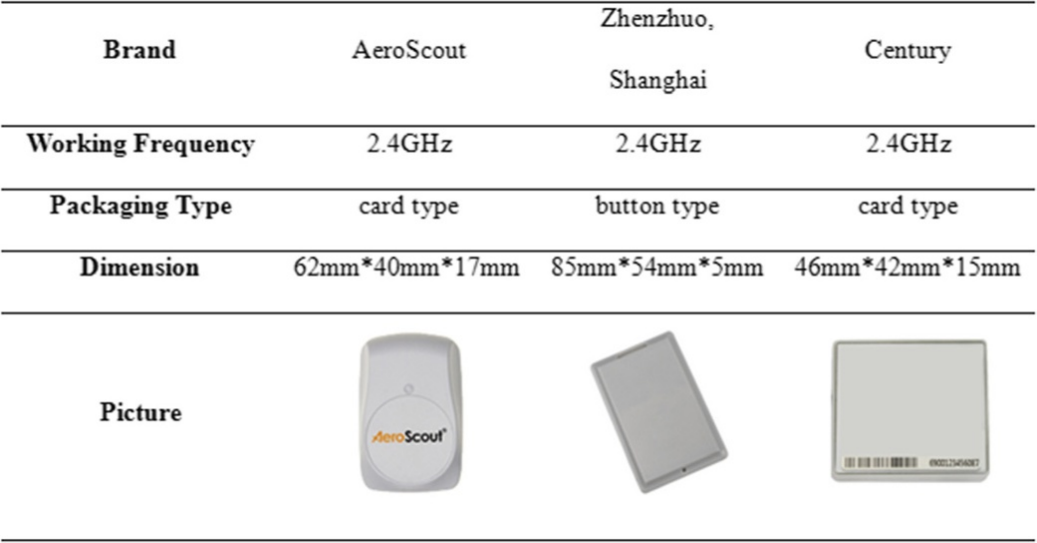
**The experimental RFID tags’ detailed parameters.** Working frequency, packaging type, dimension and picture of each experimental RFID tag brand.

The detailed parameters of the experimental RFID tags are shown in Figure 7.

### Statistical analysis

All measurement data were tested by a One-Sample Kolmogorov-Smirnov Test to verify their probability distribution, and all groups were found to have a normal distribution (*p* > 0.05).

Statistical results were expressed as mean value ± SD. For variables, comparisons between two groups were performed by using an independent samples Student’s *t* test, and comparison between multiple groups were performed using a one-way analysis of variance with the software SPSS, version 13.0 (SPSS Inc., Chicago, IL, USA). Statistical significance was accepted when *p* < 0.05. The authors had full access to and took responsibility for the integrity of the data.

## Results

All examinations were performed without any technical problems and the image quality was sufficient for data analysis in all cases.

### Calculation of the RFID tags’ usable rate

The results showed that the usability of RFID tags was not affected when being placed in high magnetic field. All the RFID tags’ identification number could be read completely and correctly regardless of the type of MR scan sequence, scan time and RFID type. The RFID tags could be recognized rapidly by the reading device without data loss or corruption. The maximum temperature difference of RFID tags after and before MR scan was 0.32°C when the distance was 5 cm. The minimum temperature difference was -0.13°C when the distance was 70 cm. No obvious heating effects were detected on any RFID tag. The usable rate is listed in Table 2 and the temperature difference is listed in Table 3.

**Table 2:** **RFID tags usable rate**

Sequence	SE		GRE		IR	
Time of duration (min)	10	30	10	30	10	30
Card type	100%	100%	100%	100%	100%	100%
Button type	100%	100%	100%	100%	100%	100%

RFID tags usable rate for different scan sequence, scan time and RFID type.

**Table 3:** **Temperature difference**

Time of duration (min)		10				30			
Distance (cm)		5	10	30	70	5	10	30	70
Temperature difference (°C)	Environment	-0.09	-0.08	-0.25	-0.31	-0.1	-0.72	-0.75	-0.9
	Card type	0.14	0.11	0.05	-0.06	0.16	0.14	0.03	-0.13
	Button type	0.31	0.12	0.01	-0.02	0.32	0.12	-0.03	-0.08

The temperature difference for different scanning time, distance and RFID type.

### Subjective evaluation of the MR image quality

**Figure 8:**
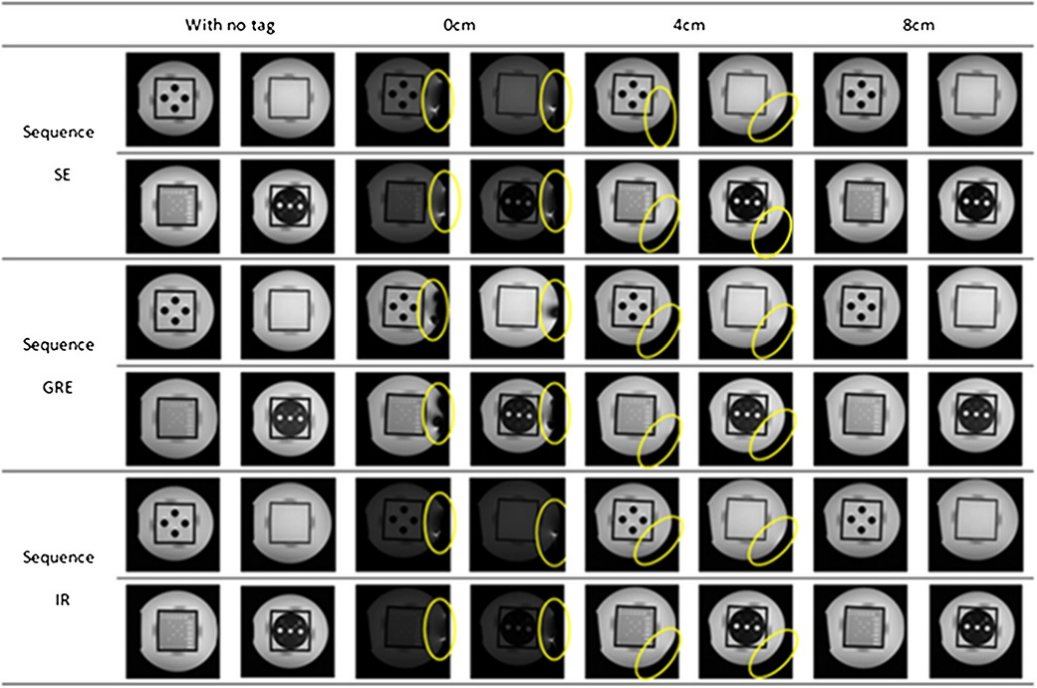
**The artifact of imaging affected by the RFID tags.** Artifacts affected by the RFID tags in each scanning distance using the three scan sequences; with no tag, control group; 0 cm, 4 cm, 8 cm, experimental group.

As Figure 8 showed, the image quality was visibly affected and artifacts could be observed in the region near to the RFID tags when they were attached tightly to the phantom in the three scan sequences. However, no visible artifacts could be observed in any of the four layers’ images when the distance between the edge of phantom and the RFID tag was 8 cm. Figure 7 illustrates that the artifact gradually disappeared with the RFID tags moving away from the MRI coil.

### Measurement of SNR

There was no significant difference in SNR between the groups with (Group 1) and without RFID tags (Group 0) in the SE and IR sequences (*p* > 0.05). However, when using the GRE sequence, the SNR of the whole image was significantly different between Group 0 and Groups 1–4 *(p <* 0.05), and the SNR of Group 1 was almost twice that of Group 0. In the meantime, there was a significant difference in SNR among the four groups (Group 1–4) with RFID tags at different distances (*p* < 0.05), showing that the SNR of images by using the GRE sequences is more sensitive to the distance to RFID. The detailed results are listed in Table 4.

**Table 4:** **Data analysis results of each scanning condition**

RFID tag	Distance	Signal-to-noise ratio			Uniformity			Geometric distortion			High contrast resolution (lp)		
		SE	GRE	IR	SE	GRE	IR	SE	GRE	IR	SE	GRE	IR
With no tag	Group 0	85.002 ± 45.11	180.5214 ± 70.32	80.6595 ± 25.37	96.34%	96.80%	96.38%	0.0131 ± 8.27E-03	0.0124 ± 7.74E-03	0.0119 ± 5.97E-03	4	4	4
With tag	Group 1 (0 cm)	82.5572 ± 39.90	78.3693 ± 50.29	75.9142 ± 24.81	96.22%	97.66%	96.00%	0.0136 ± 1.09E-02	0.0127 ± 1.25E-02	0.0122 ± 1.15E-02	4	4	4
	Group 2 (4 cm)	82.6249 ± 47.57	99.7621 ± 58.52	76.1715 ± 25.89	96.34%	97.22%	96.07%	0.0126 ± 8.73E-03	0.0131 ± 1.25E-02	0.0128 ± 9.73E-03	4	4	4
	Group 3 (8 cm)	82.7500 ± 41.37	106.6630 ± 47.95	77.8441 ± 23.31	96.41%	97.03%	96.23%	0.0133 ± 8.59E-03	0.0129 ± 1.18E-02	0.0121 ± 9.82E-03	4	4	4
	Group 4 (10 cm)	86.7404 ± 66.00	112.3880 ± 52.41	81.7903 ± 24.68	96.17%	96.87%	96.08%	0.0134 ± 1.09E-02	0.0113 ± 9.83E-03	0.0115 ± 9.72E-03	4	4	4

Data analysis results of measured SNR, uniformity, geometric distortion and high-contrast resolution in each scan distance in the three scan sequences.

### Measurement of uniformity

For SE sequence, there was a significant statistical difference between Groups 0 and 1 (*p* < 0.05), although the average data value only changed marginally. At the same time, there was no significant difference between the results of Group 0 and Group 2–4. For GRE sequence, the average uniformity value differed significantly between Group 1 and Groups 0 and 2–4 (*p* < 0.05). There was no significant difference between groups 0 and group 2, 3 and 4. For IR sequence, there was no significant difference in uniformity across the five groups (Groups 0–4). The detailed results are listed in Table 4.

### Measurement of geometric distortion

To evaluate the overall image quality more objectively, points in the area which was close to the RFID tags and where distortion could be visibly detected were not used in the Geometric Distortion calculation. The data only showed the actual distortion level of the area without visible changes.

There was no significant statistical difference in Geometric Distortion across the five groups in the three scan sequences (*p* > 0.05). However, the standard deviation of the Geometric Distortion measurements in Group 1 in the SE and IR sequences and Group 1–3 in the GRE sequence were much higher than that of Group 0 in each sequence respectively, reflecting a slight distortion of the images. The detailed data are listed in Table 4.

### Evaluation of high-contrast resolution

High-contrast resolution was evaluated directly by six examiners including three technicians and three radiologists. Consistent evaluation was provided by all examiners. The detailed data are listed in Table 4.

## Discussion

Currently, active RFID tags used in patient and medical equipment positioning and tracking are greatly encouraged in hospitals to improve management. Most RFID manufacturers claim that RFID technology is safe enough to be used in the health care environment, and that RFID tags have no effect on any other medical treatment [13–15]. Researchers at St. Gallen Canton Hospital in Switzerland reported that high-frequency 13.56 MHz RFID tags would not interfere with the functionality of imaging devices [15] significantly. However, there are reports stating that RFID technology is capable of inducing potentially hazardous incidents in medical devices, especially in implantable pacemakers, defibrillators and critical care equipments [5–8]. In this study, repetitive experiments were performed in three typical scan sequences with a phantom of standard size to investigate the effects of active RFID tags having on MR imaging quality.

The results of this study show that active RFID tags can significantly affect MR imaging quality if the distance between the scanned object and RFID tag is close. Visible artifacts could be observed if the distance was shorter than 4 cm. In the GRE sequence, the distortion was larger than that in the other two sequences. However, if the distance was longer than 8 cm, no obvious distortion could be observed then.

The quantitative indices of the MR image quality also demonstrates that RFID tags have negative impacts on MR image quality, and this impact is also related to the distance. When the distance was long enough, all the affection diminishes and the image quality returns to normal. Furthermore, the quality of images got by the GRE sequence was more sensitive to the distance to RFID than the other two sequences. That effect was evident from the reduction in SNR, which decreased to almost half when the RFID tag was close to the scanning target in GRE. Moreover, the uniformity of group 0 by GRE also changed much more than those by the other two sequences. This result is probably due to the GRE sequence which uses the gradient magnetic field to inspire the signal and thus is more sensitive to small magnetic field changes.

According to the results of this study, RFID tags can be used normally after a long-time scanning regardless of the sequence types. This observation provides very meaningful guidance for clinical workflow. If a controversial result is observed, the workflow for patients with RFID who needs MR scan will become very complicated. However, for safety reasons, the medical staffs still should give the patient another identification card before the MR scan and double check RFID tags after scan to ensure the patient can be identified properly.

The temperature of RFID tags did not rise obviously regardless of packaging types even when RFID tags were within the scan coil. In this experiment, when the distance between the phantom and the RFID tags increased to 70 cm, the final temperature was lower than initial temperature. This is because MR scan did not have obvious heating effect on RFID tags but the environment temperature decreased 1°C at that time.

The lack of studies on the safety of RFID may be because of the low number of incidents reported about hazardous incidents caused by smart phones and wireless local-area-network, which are thought to be more powerful than RFID tags [16, 17]. Although the risk of EMI affecting medical devices caused by RFID is likely small, healthcare facilities should consider the possibility of EMI when deciding how and where to use RFID technology. In this study, only the MR environment was investigated. Further studies on potential EMI incidents that may be induced by RFID on other medical devices should be performed and specific conditions under which RFID and medical devices can work together properly should be investigated.

This study has some limitation. First, the step length of distance is rather big. Thus drawing a curve relationship between imaging quality indices and distance is insufficient. We will perform further experiments with smaller distance steps. Second, our results are only applied to the technology of three active RFID systems at 2.4 GHz frequency. Although these three kinds of RFID tags are meticulously selected in the tracking patients and medical equipments and could be considered as a representative sample of RFID equipments used for applications in health care, it would be better if we could test more RFID tags made by different manufacturers with different frequencies.

## Conclusions

Active RFID tags can affect MR imaging quality, especially when a GRE sequence is used. Increasing the distance from RFID tags to the imaging target can decrease this influence. When the distance is longer than 8 cm, MR imaging quality is almost unaffected. However, a gradient echo related sequence is not recommended when patients are wearing RFID wristbands.

## Authors’ information

Xiaolu Fei, senior engineer, Information Technology Department, Xuanwu Hospital Capital Medical University, China. Shanshan Li, assistant engineer, Information Technology Department, Xuanwu Hospital Capital Medical University, China. Shan Gao, intermediate engineer, Information Technology Department, Xuanwu Hospital Capital Medical University, China. Lan Wei, intermediate engineer, Information Technology Department, Xuanwu Hospital Capital Medical University, China. Lihong Wang, vice-president, Xuanwu Hospital Capital Medical University, China.

## Abbreviations

HIT: Health information technology
RFID: Radio frequency identification
EMI: Electromagnetic interference
MR: Magnetic resonance
SE: Spin-echo
GRE: Gradient recalled echo
IR: Inversion recovery
SNR: Signal-to-noise ratio
SAR: Specific absorption rate
ROI: Region of interest
AP: Access point
IOT: Internet of things
TR: Repetition time
TE: Echo time
FOV: Field of view.
